# mRNA Vaccination for COVID-19 Lowers the Risk for Pulmonary Fibrosis Through GIP-10/Gal-3/HIF-1 Downregulation

**DOI:** 10.7759/cureus.84269

**Published:** 2025-05-17

**Authors:** Maha R Al-Sammarraie, Fatma D Azaiez, Hejer Litaiem

**Affiliations:** 1 Department of Chemistry, University of Baghdad, Baghdad, IRQ; 2 Laboratory of Inorganic Chemistry, Faculty of Sciences, University of Sfax, Sfax, TUN; 3 Faculty of Pharmacy, University of Monastir, Monastir, TUN; 4 Department of Chemistry, University of Sfax, Sfax, TUN

**Keywords:** gal-3, hif-1, inflammation, leukocytes, pfizer covid-19 vaccine

## Abstract

Background

Pulmonary fibrosis (PF) is associated with coronavirus disease 2019 (COVID-19) through the occurrence of acute respiratory distress syndrome (ARDS). The overall sequence results in the elevation of the inflammation profile of infected individuals. The risk of PF onset in COVID-19 patients is not limited to the infection course but extends to post-infection periods. Gamma-inducible protein-10 (GIP-10) is a chemokine; its production and release are induced by interferon-gamma (IFN-γ).

Objectives

In this study, we aimed to investigate the role of GIP-10, Galectin-3 (Gal-3), and hypoxia inducible factor 1 (HIF-1) in PF-associated COVID-19 and the effectiveness of the Pfizer vaccine against the progression of PF and inflammation through evaluating these three biomarkers and their correlation with a few hematological parameters.

Design & methods

The study included 120 subjects (34-68 years) from Ibn Al-Nafees Hospital (Baghdad, Iraq). Three groups of 40 subjects were designed for our investigation as control, non-vaccinated COVID-19, and vaccinated COVID-19 patients. The presence of PF was evaluated in each participant. The COVID-19 patients with chronic kidney disease, liver cirrhosis, cancer, and pregnant women were excluded from this study. The GIP-10, Gal-3, and HIF-1 were evaluated in the subjects’ serum using Sandwich ELISA technology.

Results

The results have shown significantly elevated levels of GIP-10, Gal-3, and HIF-1 in vaccinated and non-vaccinated PF-associated COVID-19 patients compared to the control, but the vaccinated patients exhibited significantly (*p*<0.05) lower levels of GIP-10, Gal-3, and HIF-1 compared to non-vaccinated patients. Moreover, in non-vaccinated PF-associated COVID-19 patients, GIP-10 did not correlate significantly with any parameter, while in vaccinated patients, it was correlated positively with age, WBC, RBC, and ESR. All of GIP-10, Gal-3, and HIF-1 expressed Odds ratios (OR) 1< as risks for PF in COVID-19 patients and can be used excellently to predict PF-associated COVID-19.

Conclusions

The Pfizer vaccine for COVID-19 has a positive role in managing GIP-10 and, therefore, better controls patients' inflammation profiles.

## Introduction

Pulmonary fibrosis (PF) is a pathological condition characterized by the replacement of healthy lung tissue with an abnormal extracellular matrix, destroying alveolar architecture. This leads to reduced lung compliance, diminished exchange of gases, and eventually respiratory failure and mortality. Individuals with PF commonly experience shortness of breath [1]. The causes of pulmonary fibrotic disorders are diverse, including a range of factors such as allergies, chemicals, radiation, and environmental agents. Nevertheless, the etiology of idiopathic pulmonary fibrosis, one of the prevailing pulmonary fibrotic diseases, remains unexplained [2]. In the past few years, during the pandemic of coronavirus disease 19 (COVID-19), the respiratory system was the major target of the virus among other organs [3]. Epidemiological studies have reported that ~3.5-17% were suffering from acute respiratory distress syndrome (ARDS), with very high rates of mortality among these patients [4, 5]. ARDS is recognized as a risk factor for the occurrence of PF due to its severe disruption of the alveolar capillary barrier. This disruption results in increased permeability, leading to the accumulation of fluid in the interstitial space and alveoli. This fluid accumulation may create a transparent membrane on the surface of the alveoli, which has the potential to progress into PF [6]. COVID-19 has been characterized by a cytokine storm that leads to elevated inflammation profiles in individuals [7]. This problem means that the risk of PF is not limited to the association with the infection course but extends into post-infection periods [8]. It is important to estimate the effectiveness of COVID-19 vaccines against the physiological alterations occurring during and post-infection time.

Gamma-inducible protein-10 (GIP-10) is a chemokine whose production and release are induced by interferon-gamma (IFN-γ) [9]. GIP-10 is expressed in various types of cells, most importantly in the airways, and acts to attract Th1 lymphocytes by binding to CXCR3 receptors that are highly expressed on their cellular surface [10]. Studies have reported elevated levels of GIP-10 associated with chronic inflammation in the respiratory system [11, 12]. In COVID-19, GIP-10 is one of the pro-inflammatory mediated peptide’s pool that responds to the virus, causing a high inflammation profile in individuals [13]. Moreover, Galectin-3 (Gal-3), a multifunctional lectin protein belonging to the family of β-galactoside-binding animal lectins, is involved in many physiological processes and pathological conditions, where it can increase the apoptotic resistance [14]. An increasing body of evidence suggests that Gal-3 facilitates the biosynthesis of collagen, stimulates the proliferation and metamorphosis of fibroblasts, and activates numerous profibrotic factors. Gal-3's crucial functions in fibrogenesis have been elucidated in multiple systems of organs, including the renal system, liver, lungs, and myocardium, in recent research [15]. Furthermore, Gal-3 is involved in acute phase inflammation and the transition into the chronic state [16]. This may indicate a crucial role for Gal-3 to play in the PF-associated COVID-19 and the residual effects of the infection and vaccine.

Hypoxia-inducible factor 1 (HIF-1) is another protein involved in inflammatory regulating events [17]. The principal role for HIF-1 is to regulate the expression of many genes during hypoxia, enabling the cell to adapt the suitable metabolic pathway in a low oxygen environment [18]. HIF-1 has been reported to induce fibrosis in kidneys [19] and is involved in the pathophysiology of PF [20]. Therefore, we attempted to investigate the role of GIP-10, Gal-3, and HIF-1 in PF-associated COVID-19 and the effectiveness of the Pfizer vaccine against the progression of PF and inflammation by evaluating these biomarkers and estimating if they have a role in the progression of PF.

## Materials and methods

Subjects

This study included the participation of 120 subjects from Ibn Al-Nafees Hospital from March 2022 to August 2022. The subjects were classified into three groups as follows:

i) Control group: Contained 40 subjects (34-68 years) with no infection at the time of sample collection, and with no presence of PF.

ii) Non-vaccinated COVID-19 group: Contained 40 subjects (40-66 years) with a confirmed infection of COVID-19 using Real-time PCR technology. These subjects had never received any dose of any known vaccine for COVID-19. Moreover, 19 subjects were confirmed to have PF by CT-scan and X-ray imaging.

iii) Vaccinated COVID-19 group: Contained 40 subjects (40-63 years) with a confirmed infection of COVID-19 using Real-time PCR technology. The subjects have received two doses of the Pfizer/BioNTech vaccine for COVID-19 before the time of sample collection. Moreover, 20 subjects were confirmed to have PF by CT-scan and X-ray imaging.

People with a history of cardiovascular diseases, chronic kidney disease, metabolic disorders, and pregnant women were excluded from this study. The ethical approval for the research was obtained from the College of Science, University of Baghdad (Ref. CSEC1042410029 on 10 April 2024).

Methods

The subjects donated 10 mL of venous blood for the study experiments, which was centrifuged (4000 rpm for 10 min) to obtain serum, which was stored in three tubes for each sample at -20 ºC until analyses. The tests that required whole blood were performed immediately without storage. Hematological parameters including white blood cell (WBC) count, red blood cell (RBC) count, and hemoglobin (Hb) were analyzed in whole blood using BC-30s Mindray (Shenzhen, China) hematological autoanalyzer system, and erythrocyte sedimentation rate (ESR) was analyzed using sodium citrate anticoagulant (BDH Chemicals, UK) in whole blood, and allowed to settle undisturbed for 1 hour at room temperature.

The levels of GIP-10, Gal-3, and HIF-1 were evaluated using an ELISA kit from Biont (China) that were based on Sandwich technology where the microplate of each test was coated with an antibody of the tested cytokine that binds that cytokine with high affinity, then a second labelled antibody added and binds the cytokine to form a sandwich immunoassay that can be detected spectrophotometrically at 450 nm. Moreover, the activities of alanine aminotransferase (ALT), aspartate transaminase (AST), and alkaline phosphatase (ALP), and the levels of urea and creatinine were determined spectrophotometrically (Apel PD-303, Apel Co., Japan) using Linear (Spain) kits.

Statistical analyses

The obtained data were analyzed for statistical values using the Statistics Package for Social Sciences (SPSS) version 26.0 (IBM Corp., Armonk, NY, USA). The normality of values distribution was analyzed using a Q-Q plot. Furthermore, the comparison of means was analyzed using analysis of variances (ANOVA), and post-Hoc least significant differences (LSD) tests, considering p-values of ≤0.05 significant. Moreover, the correlations were estimated using Pearson’s coefficient (r). The diagnostic sensitivities of GIP-10, Gal-3, and HIF-1 were estimated using receiver operating characteristics (ROC) for PF progression. Moreover, the association between GIP-10, Gal-3, and HIF-1 with the risk of PF was assessed using binary logistic regression to obtain adjusted odds ratio (OR) that was corrected for age, sex, and body mass index (BMI).

## Results

The age and BMI were non-significantly (p>0.05) different among subjects across the three groups. The count of WBC was significantly (p<0.05) higher in non-vaccinated COVID-19 patients and vaccinated COVID-19 patients compared to controls, where the count of non-vaccinated patients was also significantly (p<0.05) higher compared to vaccinated patients. RBC and Hb levels were significantly (p<0.05) higher in non-vaccinated COVID-19 patients compared to vaccinated patients and controls, where the differences in their levels were non-significant (p>0.05) between vaccinated patients and controls. ESR level was significantly (p<0.05) higher in non-vaccinated PF-associated COVID-19 patients and vaccinated COVID-19 patients compared to controls, where the ESR level of non-vaccinated patients was significantly (p<0.05) higher compared to vaccinated patients. Finally, GIP-10, Gal-3, and HIF-1 levels were increased significantly in non-vaccinated COVID-19 patients and vaccinated COVID-19 patients compared to controls, where also, the GIP-10, Gal-3, and HIF-1 levels of non-vaccinated patients were significantly (p<0.05) higher compared to vaccinated patients. The data are shown in Table 1 as mean±SD.

**Table 1:** Characteristics and parameters levels of subjects. #sig compared to control; *sig compared to non-vaccinated patients according to least significant difference (LSD) post-hoc, M: male; F: female. ALT: alanine aminotransferase; AST: aspartate transaminase; ALP: alkaline phosphatase

Parameters	Control	Non-vaccinated COVID-19 patients	Vaccinated COVID-19 patients	F	p-value
Number	40	40	40	-	-
Age (year)	50.65±9.25	53.80±7.25	51.98±5.97	1.727	0.182
Sex	22M/18F	24M/16F	22M/18F	-	-
BMI (kg/m^2^)	26.44±1.97	27.17±4.41	27.47±3.23	0.998	0.372
WBC (cell/µL)	5407.50±754.44	12335.00±1061.10 #	6400.00±672.54 *#	784.198	<0.001
RBC × 10^6^ (cell/µL)	5.15±0.60	8.24±1.24 #	5.29±0.57 *	164.053	<0.001
Hb (g/dL)	14.31±1.33	10.31±0.76 #	14.25±1.61 *	154.116	<0.001
ESR (mm/h)	13.25±3.24	24.34±3.52 #	15.40±2.12 *#	85.202	<0.001
GIP-10 (ng/mL)	97.52±4.40	182.33±27.73 #	144.90±18.47 *#	191.957	<0.001
Gal-3 (pg/mL)	2.67±0.80	93.01±24.16 #	43.66±11.17 *#	346.179	<0.001
HIF-1 (ng/mL)	7.02±0.83	25.68±9.20 #	14.59±3.85 *#	105.465	<0.001
ALT (IU/mL)	6.65±1.44	39.81±1.90 #	11.17±1.14 *#	5554.477	<0.001
AST (IU/mL)	10.25±1.08	36.53±6.02 #	15.56±1.64 *#	577.720	<0.001
ALP (IU/mL)	53.58±6.67	173.66±13.68 #	87.95±9.01 *#	1466.601	<0.001
Urea (mg/dL)	13.51±3.89	29.56±4.79 #	20.88±3.23 *#	159.612	<0.001
Creatinine (mg/dL)	0.88±0.20	4.06±0.76 #	1.55±0.28 *#	483.199	<0.001

In non-vaccinated COVID-19 patients, GIP-10 did not correlate with any parameter, while in vaccinated patients, it was correlated positively and weakly with age, positively and moderately with WBC, positively and weakly with RBC and ESR, and negatively and weakly with Gal-3 (Table 2). Gal-3, on the other hand, correlated positively and moderately with WBC and HIF-1 in non-vaccinated patients and with Hb and GIP-10 in vaccinated patients. Finally, HIF-1 was correlated with Gal-3 in non-vaccinated patients and RBC and ESR in vaccinated patients (Table 2).

**Table 2:** Correlation of GIP-10, Gal-3, and HIF-1 in PF-associated COVID-19 patients. * represents significant correlation.

Parameters	Non-Vaccinated patients						Vaccinated patients					
	GIP-10		Gal-3		HIF-1		GIP-10		Gal-3		HIF-1	
	r	p	r	p	r	p	r	p	r	p	r	p
Age (year)	-0.093	0.569	0.255	0.113	0.169	0.296	0.353*	0.025	0.188	0.246	-0.023	0.886
BMI (kg/m^2^)	-0.043	0.791	0.030	0.854	0.135	0.408	-0.250	0.120	-0.050	0.757	0.043	0.791
WBC (cell/μL)	-0.008	0.962	-0.413*	0.008	-0.128	0.432	0.508*	0.001	0.011	0.947	-0.141	0.387
RBC (cell/μL)	0.136	0.402	0.257	0.109	0.001	0.998	0.393*	0.012	0.146	0.368	0.333*	0.036
Hb (g/dL)	0.289	0.071	0.143	0.380	-0.147	0.367	-0.296	0.064	0.687*	<0.001	0.246	0.126
ESR (mm/h)	0.275	0.086	-0.188	0.245	-0.086	0.597	0.327*	0.039	0.214	0.185	0.336*	0.034
GIP-10 (ng/mL)	-	-	-0.059	0.716	-0.150	0.355	-	-	-0.337*	0.033	-0.303	0.057
Gal-3 (pg/mL)	-0.059	0.716	-	-	0.557*	<0.001	-0.337*	0.033	-	-	0.300	0.060
HIF-10 (ng/mL)	-0.150	0.355	0.557*	<0.001	-	-	-0.303	0.057	0.300	0.060	-	-

**Figure 1:**
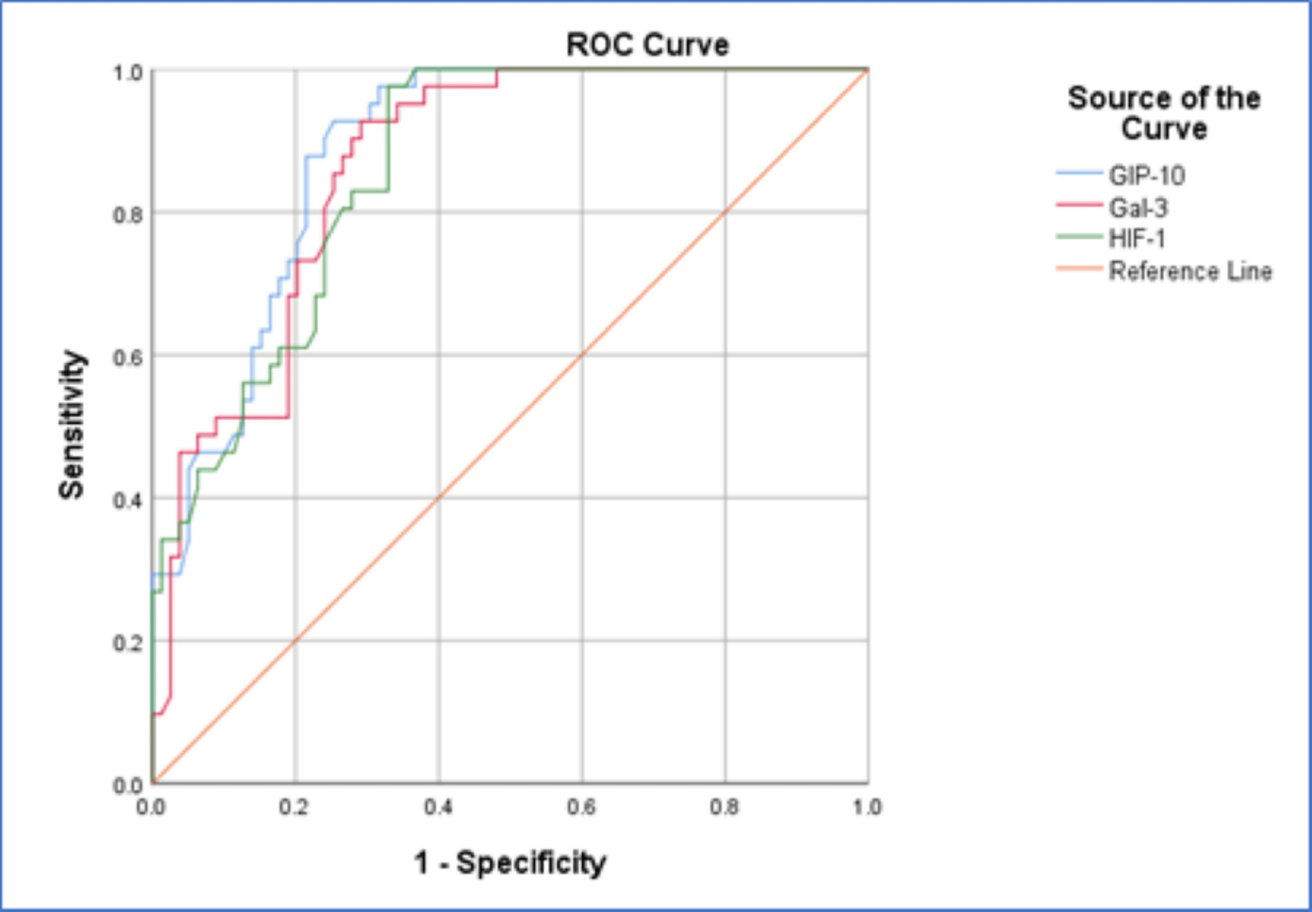
The ROC curve of GIP-10 (A), Gal-3 (B), and HIF-1 (C) in the prognosis of PF-associated COVID-19.

In the prognostic evaluation of GIP-10, it has shown a good sensitivity toward PF-associated COVID-19 prognosis with the area under the curve (AUC) of 0.885 and a cut-off value of 149.60 ng/mL in 87.8% sensitivity and 78.5% specificity (Figure 1). Gal-3 has also shown good sensitivity in the prognosis of PF-associated COVID-19 with an AUC of 0.864 and a cut-off value of 49.775 pg/mL in 85.4% sensitivity and 74.7% specificity (Figure 1). Finally, HIF-1 has shown good sensitivity in the prognosis of PF-associated COVID-19 with an AUC of 0.861 and a cut-off value of 15.0 ng/mL in 82.9% sensitivity and 72.2% specificity (Figure 1, Table 3).

**Table 3:** ROC curve parameters for GIP-10, Gal-3, and HIF-1.

Parameter	GIP-10 (ng/mL)	Gal-3 (pg/mL)	HIF-1 (ng/mL)
AUC	0.885	0.864	0.861
SE	0.029	0.032	0.032
p-value	<0.001	<0.001	<0.001
Cut-off value	149.60	49.775	15.0
Sensitivity	87.8%	85.4%	82.9%
Specificity	78.5%	74.7%	72.2%
95% CI	0.828-0.941	0.801-0.927	0.798-0.924

The risks were determined by using logistic regression for GIP-10, Gal-3, and HIF-1 (Table 4). GIP-10 has shown a strong association with COVID-19 (OR=1.550, CI at 95% 1.241-1.935), thus, an increase in 1 ng/mL of GIP-10 level corresponds to a 55% increase in the probability of being PF-associated COVID-19. A strong association between Gal-3 and COVID-19 was observed in COVID-19 patients (OR=12.644, CI at 95% 9.827-16.268); thus, an increase in the level of Gal-3 by 1 pg/mL results in an increase in the probability of PF association during COVID-19 infection by 12.644-fold. Furthermore, HIF-1 has shown a strong association with COVID-19 infection (OR=18.272, CI at 95% 3.719-89.767); thus, an increase in the level of HIF-1 by 1 ng/mL leads to an increase in the probability of PF-associated COVID-19 infection by 18.272-fold (Table 4).

**Table 4:** Biomarkers related to the risks of PF in COVID-19 patients. OR: adjusted odds ratio; CI: confidence interval; PF: pulmonary fibrosis

Parameters	OR	CI at 95%	p-value
GIP-10	1.550	1.241-1.935	<0.001
Gal-3	12.644	9.827-16.268	<0.001
HIF-1	18.272	3.719-89.767	<0.001

## Discussion

The common target of SARS-CoV-2 is the lungs and airway duct, where the most common features are cough, fever, headache, and hypoxia [21, 22]. The virus triggers the production and release of a wide spectrum of pro-inflammatory factors in a process called a cytokine storm. As a result, the inflammation profile is enhanced and affects organs and tissues, causing alteration in the normal physiology of the body [23]. ARDS comprises a high portion of the COVID-19 consequences, which may progress and develop into PF in severe cases and over a prolonged time [24]. GIP-10 is expressed in many tissues, including the alveoli of the lung, to attract the Th1 lymphocytes into the site of infection, where these immunological cells participate in raising the inflammation profile [25]. Mulla et al. have reported a significant increase in serum GIP-10 levels in COVID-19 patients, which corresponded with the severity of the disease, indicating a role played by GIP-10 in the progression of COVID-19 [13]; similar results were reported by Tegethoff et al. [26]. Samaras et al. have reported that GIP-10 has shown the best prognostic sensitivity in the detection of severe respiratory failure onset in COVID-19 patients among other prognostic biomarkers, including soluble urokinase plasminogen activator receptor [27], which was observed similarly in this study (Figure 1). Haroun et al. investigated GIP-10 in the serum of Egyptian COVID-19 patients. Authors recorded significantly higher levels of serum GIP-10 in COVID-19 patients, where they also observed a significantly higher level of GIP-10 in critical and severe conditions compared to mild and moderate conditions of COVID-19. Moreover, they reported significantly higher counts of WBC in a similar way to the GIP-10 with a significant correlation between them [28]. In our study, we did not observe a correlation between GIP-10 and WBC. Nevertheless, GIP-10 was correlated with WBC, RBC, and ESR in vaccinated PF-associated COVID-19 patients. Shen et al. have reported a significant reduction of WBC and GIP-10 levels after receiving the AstraZeneca COVID-19 vaccine within 1-7 days of vaccination. They have reported an improvement in the inflammation profile with a reduction of the cytokine storm caused by the vaccine [29]. Karimabad et al. documented a potential role of the Pfizer vaccine on cardiovascular health in Iranian people [30]. An early and temporary inflammatory cytokine response characterized by elevated levels of IFN-γ, GIP-10, IL-6, and C-reactive protein was detected on day 2 after receiving the BNT162b2 mRNA (Pfizer/BioNtech) COVID-19 vaccine. By day 8, these levels had recovered to their initial baseline [30, 31]. They have linked these fluctuations in chemokine/cytokine profile to increased risk of coronary heart disease [30]. It has been reported that mRNA vaccines such as Pfizer/BioNtech can improve the immune memory and activation of T cells by raising the levels of interleukins, tumor necrosis factor, and GIP-10 on the first dose of vaccination [32]. In the present study, the vaccinated patients received two doses of the vaccine before the infection. Therefore, memory may contribute to the significantly lower levels of GIP-10 that were recorded in vaccinated patients compared to non-vaccinated patients (Table 1). Yet, the low study population is still a limitation for this study, as well as the long-term monitoring for patients.

The levels of Gal-3 and HIF-1 were increased in non-vaccinated patients but decreased in vaccinated patients (Table 1). Gajovic et al. indicated that Gal-3 is linked with the severity of COVID-19, where patients with critical symptoms expressed the highest level of Gal-3 raised via the overexpression of the biomarker. They have shown a positive correlation between Gal-3 and uncontrolled immune response in severe and critical patients [33]. We have found a positive correlation between Gal-3 and HIF-1 in non-vaccinated patients and a negative correlation with WBC. Sigamani et al. reported a Gal-3 antagonist treatment for viral load in COVID-19 patients. Authors have indicated that receiving a Gal-3 antagonist by COVID-19 patients led to a significant reduction in the viral load with full disappearance of the symptoms in most of the studied subjects, which is managed by inhibiting SARS-CoV-2 entry into cells through the inhibition of Gal-3 [34]. We have observed a similar effect using the Pfizer-BioNTech vaccine in COVID-19 patients with PF, where the level of Gal-3 was reduced significantly. It has been documented that a combination of vaccines with Gal-3 inhibition would result in rapid viral clearance and improve the treatment of COVID-19 [35]. Additionally, we observed a positive correlation of Gal-3 with WBC and ESR and a negative correlation of Gal-3 with GIP-10 in vaccinated patients, which can be attributed to the well-controlled immune response in patients. Gal-3 was shown to impact the generation of reactive oxygen species, the expression of NADPH oxidase enzyme, and redox signaling, all of which have been demonstrated to have a role in inflammatory processes and the development of pulmonary fibrosis [36]. Hence, the reduction of Gal-3 in vaccinated patients might reveal an anti-fibroproliferative state despite the presence of SARS-CoV-2. HIF-1 was reported to induce pulmonary hypertension in PF mice models. Bryant et al. indicated that genetically modified mice with deficient vascular HIF have shown to be protected against pulmonary hypertension [20]. Thus, PF-associated COVID-19 patients with elevated HIF-1 can develop pulmonary hypertension. Jahani et al. reported that increased HIF-1 in COVID-19 patients was linked to hypoxia and inflammatory conditions in patients [37]. In our study, a positive correlation was observed between HIF-1 and Gal-3 in non-vaccinated PF-associated COVID-19 patients, which confirms the association of HIF-1 in the inflammatory events encountered in these patients but not in vaccinated patients. Additional studies are required to confirm the effect of vaccination on PF development and progression in COVID-19 patients.

## Conclusions

The Pfizer-BioNTech vaccine for COVID-19 has been shown to reduce the inflammation profile in PF-associated COVID-19 patients by reducing the levels of GIP-10, Gal-3, HIF-1, WBC, and ESR. This can give good control of the immune response in COVID-19, improving their symptoms and reducing the health consequences of the disease. Further investigations are required, especially, the PF progression can be observed after a long time from the infection.
